# A point mutation in *cpsE* renders *Streptococcus pneumoniae* nonencapsulated and enhances its growth, adherence and competence

**DOI:** 10.1186/s12866-014-0210-x

**Published:** 2014-08-28

**Authors:** Thierry O Schaffner, Jason Hinds, Katherine A Gould, Daniel Wüthrich, Rémy Bruggmann, Marianne Küffer, Kathrin Mühlemann, Markus Hilty, Lucy J Hathaway

**Affiliations:** Institute for Infectious Diseases, University of Bern, Friedbühlstrasse 51, CH-3010 Bern, Switzerland; Graduate School for Cellular and Biomedical Sciences, University of Bern, Bern, Switzerland; Bacterial Microarray Group at St George’s (BμG@S), Division of Clinical Sciences, St George’s, University of London, London, United Kingdom; Interfaculty Bioinformatics Unit, University of Bern, Bern, Switzerland; Swiss Institute of Bioinformatics, Lausanne, Switzerland; Department of Infectious Diseases, University Hospital of Bern, Bern, Switzerland

**Keywords:** *Streptococcus pneumoniae*, *cpsE*, Capsule, Nonencapsulated, SNP

## Abstract

**Background:**

The polysaccharide capsule is a major virulence factor of the important human pathogen *Streptococcus pneumoniae*. However, *S. pneumoniae* strains lacking capsule do occur.

**Results:**

Here, we report a nasopharyngeal isolate of *Streptococcus pneumoniae* composed of a mixture of two phenotypes; one encapsulated (serotype 18C) and the other nonencapsulated, determined by serotyping, electron microscopy and fluorescence isothiocyanate dextran exclusion assay.

By whole genome sequencing, we demonstrated that the phenotypes differ by a single nucleotide base pair in capsular gene *cpsE* (C to G change at gene position 1135) predicted to result in amino acid change from arginine to glycine at position 379, located in the cytoplasmic, enzymatically active, region of this transmembrane protein. This SNP is responsible for loss of capsule production as the phenotype is transferred with the capsule operon. The nonencapsulated variant is superior in growth *in vitro* and is also 117-fold more adherent to and more invasive into Detroit 562 human epithelial cells than the encapsulated variant.

Expression of six competence pathway genes and one competence-associated gene was 11 to 34-fold higher in the nonencapsulated variant than the encapsulated and transformation frequency was 3.7-fold greater.

**Conclusions:**

We identified a new single point mutation in capsule gene *cpsE* of a clinical *S. pneumoniae* serotype 18C isolate sufficient to cause loss of capsule expression resulting in the co-existence of the encapsulated and nonencapsulated phenotype. The mutation caused phenotypic changes in growth, adherence to epithelial cells and transformability. Mutation in capsule gene *cpsE* may be a way for *S. pneumoniae* to lose its capsule and increase its colonization potential.

**Electronic supplementary material:**

The online version of this article (doi:10.1186/s12866-014-0210-x) contains supplementary material, which is available to authorized users.

## Background

The Gram positive bacterium *Streptococcus pneumoniae* frequently colonizes the nasopharynx but can also invade the host causing serious illnesses such as pneumonia, meningitis or bacteraemia [1]. A principal virulence factor of *S. pneumoniae* is the polysaccharide capsule protecting it from host immune defences by interfering with the deposition of complement and therefore opsonophagocytosis [2-4]. The capsule is the target of all currently available pneumococcal vaccines including the 13-valent pneumococcal conjugate vaccine (PCV13) for children. The biochemical structure and linkage of repeating polysaccharide subunits determines the serotype of encapsulated strains. So far, more than 90 different serotypes have been identified [5-11] which differ in the type and number of genes encoding the proteins responsible for transcription, polymerization, elongation and export of the capsule. For almost all serotypes the capsule-encoding operon is located between non-capsule genes *dexB* and *aliA* [6,12,13]. The first four genes *cpsA*, *cpsB*, *cpsC* and *cpsD* are thought to play a role in regulation of capsular production and are largely conserved between serotypes [14,15].

Despite the importance of the capsule as a virulence factor, nonencapsulated pneumococci occur and in the nasopharynx may represent around 15% of pneumococcal isolates [16]. Nonencapsulated pneumococci are generally considered not to be virulent but are associated with outbreaks of conjunctivitis [17-19].

Although lacking the protection from opsonophagocytosis which a capsule affords, the absence of capsule may confer advantages. Nonencapsulated pneumococci are less sensitive to α-defensins, elastase and cathepsin G of neutrophils, possibly due to the difference in their surface charge compared to encapsulated pneumococci [16,20]. Conversely, capsule might reduce agglutination by mucus, increasing access to epithelial cells and so aiding colonization, at least in mice [21] and may contribute to antibiotic tolerance [22].

However, laboratory-generated nonencapsulated mutants have shown that possession of a capsule is a burden for growth [23]. For pneumococci which do have a capsule, downregulation of its expression in response to the environment helps colonization by aiding adherence to respiratory epithelial cells [24].

Nonencapsulated *S. pneumoniae* may be divided into two groups: those which have *aliB*-like homologues or *nspA* gene in place of capsule genes and those which have a capsule operon very similar to that of an encapsulated strain [25-27]. For the latter, loss of capsule expression may be due to point mutations in capsule genes or spontaneous, reversible sequence duplication or non-reversible deletion within the capsule operon as described for serotypes 3, 8, 19F and 37 [28-33]. In the laboratory, nonencapsulated variants can be obtained by knocking out specific genes of the capsule operon. D39 mutants lacking capsule genes *cps2K*, *cpsJ* or *cps2H* required suppressor mutations in *cpsE* (also denoted as *wchA*) to survive [34,35]. CpsE is the initial glycosyltransferase enzyme that catalyzes the transfer of the activated glucose-phosphate to the lipid carrier [36-40]. Previous research has shown that a functional CpsE protein is essential for encapsulation of pneumococci serotypes 9N, 13, 14, 15B and 19F [12,37,41].

During our studies of nasopharyngeal clinical isolates of pneumococci we observed an isolate which gave a mixture of larger smooth colonies (serotype 18C) and smaller rough colonies. We aimed to discover whether this was due to the presence of encapsulated and nonencapsulated versions of the same strain and, if so, to uncover the mechanism of the loss of capsule expression. We compared the two phenotypes in terms of growth, adherence to epithelial cells and competence for genetic transformation.

## Methods

### Bacterial strains

*Streptococcus pneumoniae* strain 307.14 (MLST 113) was isolated in Switzerland from the nasopharynx of a child with otitis media and determined to be serotype 18C by the Quellung reaction as previously described [25,42].

A single colony from the nasopharyngeal swab was cultured in broth once before freezing the stock. Plating out of this stock showed that there were two 307.14 variants (encapsulated, nonencapsulated) which were purified by three consecutive passaging steps where each time one single colony was picked and streaked on a Columbia sheep blood agar (CSBA) plate. Separation was confirmed by serotyping and FITC-dextran exclusion assay (data not shown).

Serotyping was performed by Quellung reaction with serotype-specific antisera from the Statens Serum Institute (Copenhagen, Denmark).

Restriction fragment length polymorphism (RFLP) analysis was performed as described previously [43], with the following modifications, to confirm that the two phenotypes had the same genetic background. The initial denaturation step was performed for 4 minutes at 95°C. Denaturation temperature was 95°C in the 30 cycles of PCR. Each reaction was performed in a total volume of 50 μl with 3 units FastStart Taq DNA polymerase, 200 μM deoxynucleoside triphosphates, 5 μl 10× PCR reaction buffer (without MgCl_2_) and 2 mM MgCl_2_ (all from Roche, Switzerland), 1 μM of each primer and 100 ng genomic DNA. Endonucleases AflIII, ApoI, DdeI and MseI (New England Biolabs, MA, USA) were used for digestion of the PCR product according the manufacturer’s instructions.

### Antibiotic susceptibilities

Etest® strips (AB BIODISK, Solna, Sweden, distributed in Switzerland by bioMérieux) were used to determine the minimal inhibitory concentrations (MIC) for the different antibiotics according to current international recommendations (www.clsi.org). A sterile cotton swab was soaked in 0.5 McFarland of bacterial culture and then streaked on agar plates (Mueller Hinton with 5% sheep blood). Ten minutes later, the Etest® strips were applied on the agar plates which were then incubated for 24 h and 48 h at 37°C with 5% CO_2_ atmosphere.

### Construction of revertant mutant strains

Capsule switch mutant strains were generated for both the encapsulated and the nonencapsulated 307.14 wild type variant using a Janus cassette based on the published method [23]. As a first step, a Janus mutant was made from each of the wild type phenotypes. Next, the Janus mutant (nonencapsulated) derived from the encapsulated wild type was transformed with DNA from the nonencapsulated wild type strain to create the mutant 307.14 cap-. Also, the Janus mutant derived from the nonencapsulated wild type was transformed with DNA from the encapsulated wildtype strain to create the mutant 307.14 cap+. Wild type and mutant strains used in this study are listed in Table 1 and the amplification and sequencing primers are listed in Additional file 1: Table S1. Pneumococcal strain AmiA9 (a kind gift of Regine Hakenbeck, University of Kaiserslautern, Germany) that harbours the genotype *rpsL*_K56T_ conferring streptomycin resistance [44] served as template for *rpsL*_K56T_ amplification. PCR products were purified using the Wizard® SV Gel and PCR Clean-Up system (Promega, USA). Stocks of competent recipient 307.14 variants were prepared by growing them in brain heart infusion broth (BHI) (Becton Dickinson, USA) supplemented with 5% fetal bovine serum (FBS) (Merck, Germany) to mid-logarithmic phase (optical density (OD_600nm_) = 0.5–0.8) followed by a 1:20 subculture in tryptic soy broth (TSB) (Becton Dickinson), pH 7 [45] to OD_600nm_ = 0.13. Bacteria were harvested by centrifugation at +4°C and resuspended in TSB, pH 8 + 15% glycerol (Sigma, USA) for storage at -80°C until use. DNA was extracted using the QIAamp® DNA Mini Kit (Qiagen, Germany) following the manufacturer’s instructions. Transformation was based on a previous description [46] diluting 100 μl of thawed competent bacteria in 900 μl fresh TSB pH 8 supplemented with 200 ng competence stimulating peptide (CSP-1, PolyPeptide Laboratories, Strasbourg, France). Bacteria were cultured for 10 min at 37°C before 500 ng *rpsL*_K56T_ PCR product, 1 μg *dexB-Janus-aliA* PCR product or 2 μg genomic DNA was added and the samples incubated 20–40 min at 30°C to induce competence fully, followed by 120 min incubation at 37°C. Serial dilutions made in phosphate-buffered saline (PBS), pH 7.4 were spread onto CSBA plates containing 300 μg/ml streptomycin and 500 μg/ml kanamycin and incubated at 37°C with 5% CO_2_ atmosphere overnight. Single colonies were subcultured on antibiotic selective CSBA plates prior to genomic DNA extraction and strain preservation at -80°C (Technical Service Consultants Ltd., Heywood, UK). The serotype of the clinical isolates, the Janus mutants and the capsule switch mutants was confirmed by the Quellung reaction after transformation. Insertion of the Janus cassette and replacement and correct insertion of the donor capsule was confirmed by four control PCR (see Additional file 1: Table S1) using the iProof polymerase (Bio-Rad, USA). In order to confirm successful transfer of *cpsE* wt and *cpsE* mutated version, the PCR product was sequenced by Sanger sequencing. In addition, PCR and sequencing was also performed at the sites of 6 other SNPs found to differ in the wild type phenotypes to check that these were not transferred.

**Table 1:** **Wild type and mutant pneumococcal strains used**

**Strain**	**Serotype**	**Origin/comment**
307.14 encapsulated	18C	Nasopharyngeal isolate
307.14 nonencapsulated	nonencapsulated	Nasopharyngeal isolate
307.14Δ*cps::*Janus	nonencapsulated	Laboratory mutant: strain 307.14 encapsulated which has had its capsule operon replaced by a Janus cassette
307.14 cap+	18C	Capsule switch mutant: 307.14 nonencapsulated which has had its capsule operon replaced by that of 307.14 encapsulated
307.14 cap-	nonencapsulated	Capsule switch mutant: 307.14 encapsulated which has had its capsule operon replaced by that of 307.14 nonencapsulated

### Quantification of capsule

#### Fluorescence isothiocyanate (FITC)-dextran exclusion assay

Capsule thickness was determined using fluorescence labeled dextran (2 000 kDa, Sigma) based a published method [47,48]. Bacteria were cultured in 10 ml Lacks [49-51], 20 mM glucose to OD_600nm_ = 0.5, centrifuged at 3000 × g for 5 min at room temperature, washed once with 10 ml of chemically defined medium (CDM) (no sugars) and then resusupended in 10 ml CDM (no sugars). 800 μl were subcultured in 20 ml CDM, pH 7, 5.5 mM glucose and grown to OD_600nm_ = 0.25. The bacteria were harvested by centrifugation and the pellet resuspended in 850 μl pre-chilled phosphate-buffered saline (PBS), pH 7.4. Bacterial FITC-dextran samples were prepared and visualized using a 100× objective as described [23].

The zone of exclusion of FITC-dextran indicates the polysaccharide capsule thickness. The experiment was repeated three times on different days and a total of 15 pictures of the mixed culture (307.14 encapsulated and 307.14 nonencapsulated) were taken. The serotype was confirmed by Quellung reaction.

#### Electron microscopy

Bacteria were cultured as described above for the FITC-dextran exclusion assay, grown to OD_600nm_ of 0.2–0.25 in CDM, pH 7, 5.5 mM glucose and harvested by centrifugation. Serotype was confirmed by Quellung reaction after overnight incubation at 37°C with 5% CO_2_ atmosphere on CSBA plates.

Bacteria were cryopreserved by high-pressure freezing as described before [52]. Acetone containing 2% osmium tetroxide, 0.1% uranyl acetate, 0.2% ruthenium hexamine trichloride (RHT) and a total of 4% H_2_O served as medium for freeze substitution. The RHT added improves capsule resolution [53]. Electron micrographs from cross-sectional bacterial preparations were taken at a magnification of 53 000×. The polysaccharide capsule thickness was measured perpendicular to the bacterial cell wall from at least 30 randomly selected bacterial cell bodies in 15 pictures using the free software ImageJ v1.45 l (National Institutes of Health, USA, http://imagej.nih.gov/ij). One to four measurements were taken at distinct positions of a given cell body.

#### Growth assays

Strains were streaked onto CSBA plates and incubated at 37°C in 5% CO_2_ overnight and then subcultured in the semi-defined, nutritionally relatively rich Lacks medium [49-51] supplemented with 20 mM glucose and with the following modifications: 14.7 mM C_2_H_3_NaO_2_ · 3H_2_O, 5.41 μM CaCl_2_, 0.89 μM MnSO_4_ · H_2_O (all Merck, Germany) and ≥ 12 800 U catalase (Sigma, C40) per liter Lacks medium, no NaC_2_H_3_O_2_ and no bovine albumin. For growth assays, CDM [54] representing a nutritionally limited environment was used. Since pH may affect growth and competence, CDM was stabilized using Sørensen buffer (KH_2_PO_4_, Na_2_HPO_4_ · 2H_2_O), pH 7 instead of double-distilled water (Additional file 1: Table S2). Half-loopfuls of colonies were used to inoculate 10 ml Lacks supplemented with 20 mM glucose. The bacteria were grown to OD_600nm_ of 0.5 and frozen at -80°C in aliquots in 15% glycerol. Thawed bacterial suspensions were diluted in PBS pH 7.4 and plated on CSBA to determine the number of colony forming units (CFU) per ml the next day. The serotype was confirmed by Quellung reaction. For growth assays an inoculum of 5 × 10^7^ CFUs was used for subculture in 20 ml CDM, 5.5 mM glucose. Bacteria were grown for 10 hours at 37°C in a water bath and the OD_600nm_ measured every 30 minutes. Growth assays were repeated on three different days.

#### Transformation frequency

To compare transformation frequencies between the two phenotypes the bacteria were cultured as described for the FITC-dextran exclusion assay and grown to OD_600_ = 0.15 in CDM, 5.5 mM glucose, pH 7. 0.5 ml of the culture were transferred to 9.5 ml TSB competence medium pH 8.0 prewarmed to 30°C and incubated for 15 min at 30°C. CSP-1 was added to final concentration of 100 ng/ml and the culture incubated for 15 min at 30°C. 1 μg of chromosomal DNA from streptomycin resistant strain 104.37 (serotype 6B) was added and the culture incubated for 60 min at 30°C, then for 120 min at 37°C. Serial dilutions (1:20) in PBS pH 7.4 were plated onto CSBA plates with and without 300 μg/ml streptomycin. After overnight incubation the number of colonies was counted and the transformation frequency calculated. The serotype was confirmed by Quellung reaction.

#### Adherence to and invasion of human epithelial cell line Detroit 562

Detroit nasopharyngeal epithelial cells (ATCC-CCL-138) were cultured as described [55] in 1× minimum essential medium (MEM) containing 2 mM L-glutamine, 8.9 mM sodium bicarbonate, 1 × MEM non-essential amino acids, 1 mM sodium pyruvate (all Gibco by Life Technologies, USA), 10% heat-inactivated fetal bovine serum (FBS) (Merck), 100 U/ml penicillin and 100 μg/ml streptomycin and grown until reaching complete confluence at 37°C in 5% CO_2_. For adherence and invasion assays, 500 μl culture medium (no antibiotics) with 3 × 10^5^ cells was added per well of a 24-well tissue culture plate and incubated for 24 h. *S. pneumoniae* was grown as described for the FITC-dextran exclusion assay in CDM, 5.5 mM glucose, pH 7 to mid- logarithmic phase (OD_600nm_ = 0.15 for 307.14, encapsulated and OD_600_ = 0.25 for 307.14, nonencapsulated) and 500 μl cell culture medium (no FBS or antibiotics) with 0.9 × 10^7^ bacteria were added to each well containing previously washed cells (0.85% NaCl). The 24-well plate was centrifuged at 423 × g for 5 minutes at room temperature. After incubation for 30 min at 37°C with 5% CO_2_, the cells were washed five times with saline to remove non-adherent bacteria and trypsinized with 200 μl 0.05% trypsin-EDTA (Gibco by Life Technologies). To determine the number of invasive bacteria, the gentamicin protection assay described earlier was followed and the cells co-cultured with bacteria for 3 h at 37°C with 5% CO_2_ [55,56]. The cells were washed five times with saline and 1 ml fresh MEM with gentamicin sulfate salt (200 μg/ml, Sigma) was added to each well for a 2 h-incubation at 37°C to kill extracellular bacteria. After washing with saline and trypsinization as described above, the cells were lysed by addition of 1% saponin (Sigma) and incubation for 7 minutes at room temperature. Appropriate dilutions in PBS, pH 7.4 were plated onto CSBA plates and incubated overnight. The number of colony-forming units (CFUs) was determined using an automated colony counter [57]. Adherence and invasion potential of the bacteria was calculated as the proportion of recovered bacteria to the inoculum. The serotype was confirmed by Quellung reaction.

### Whole genome analysis of bacterial genomes

#### Whole genome sequencing

A barcoded fragment library with 400–500 bp insert size using “TruSeq DNA TruSeq DNA Sample Preparation Kit” (Illumina Inc., USA) was prepared for both bacterial genomes. The samples were sequenced in one lane of an Illumina HiSeq 2000 (Illumina Inc.) together with 23 unrelated barcoded samples. This resulted in 10,276,620 paired-end reads (2 × 100 bp) for sample 307.14, encapsulated and 8,715,247 paired-end reads (2 × 100 bp) for sample 307.14, nonencapsulated.

#### *De novo* assembly

The reads of the variants 307.14 nonencapsulated and 307.14 encapsulated were subjected to *de novo* assembly using SPAdes (version 2.4.0, kmer sizes = 33,55,67,81,91,93,95,97,99) [58]. Only scaffolds equal or longer than 500 bp were used for the further analyses. The assembly of 307.14 nonencapsulated resulted in 2088272 bp in 63 scaffolds and a n50 of 79979 bp. The assembly of 307.14 encapsulated resulted in 2083495 bp in 69 scaffolds and a n50 of 71589 bp.

#### Polymorphisms detection

To detect assembly errors, for the assemblies of the strains 307.14 nonencapsulated and 307.14 encapsulated a remapping was performed using bowtie2 (version 2.0.0beta6, options: -N 1 –very-sensitive) [59]. Differences were detected using samtools (version 0.1.19, mpileup). To detect polymorphisms between the two strains, the reads of 307.14 nonencapsulated were mapped to the *de novo* assembly 307.14 encapsulated and *vice versa*. The mapping was performed using bowtie2 (version 2.0.0beta6, options: -N 1 –very-sensitive). Subsequently, polymorphisms of both mappings were determined using samtools (version 0.1.19, mpileup) [60].

### Gene expression assays

#### Microarray

Bacteria were cultured as described for the adherence and invasion assay to mid-logarithmic phase in CDM, 5.5 mM glucose, pH 7. Double volume of RNAprotect® bacteria reagent (Qiagen, Germany) was added to the bacterial suspension to stop further transcription. The samples were vortexed, incubated for 5 min at room temperature and then centrifuged at 4500 × g for 10 min at +4°C. The RNA was extracted with the RNeasy® Mini Kit (Qiagen) following the manufacturer’s instructions using a Mickle vibratory tissue disintegrator (Mickle Laboratory Engineering Company Ltd., UK) for mechanical disruption of the bacteria. Contaminating DNA was removed using the DNA-*free*™ Kit (Life Technologies) as described by the manufacturer. RNA purity, concentration and quality/integrity were checked using with the NanoDrop® spectrophotometer ND-1000 (Thermo Scientific, USA) and the RNA Nano 6000 kit for the Agilent 2100 bioanalyzer (Agilent Technologies, USA) following the manufacturer’s instructions. The entire transcriptome was analyzed by microarray as follows. RNA samples were hybridised to the BμG@S SPv1.4.0 microarray designed by the Bacterial Microarray Group at St. George’s, University of London and manufactured on the Agilent SurePrint platform (Agilent Technologies). Labelled cDNA was prepared from 1 μg total RNA using Cy3-dCTP (GE Healthcare, UK) and SuperScript II reverse transcriptase with random hexamer primers (Life Technologies). Labelled cDNA was purified by Qiagen MinElute column, combined with 10 × CGH blocking agent and 2 × Hi-RPM hybridisation buffer (Agilent Technologies) and heated at 95°C for 5 min prior to loading onto microarray slides which were incubated overnight in an Agilent rotating oven at 65°C, 20 rpm. After hybridization, slides were washed for 5 min at room temperature with CGH Wash Buffer 1 and 1 min at 37°C with CGH Wash buffer 2 (Agilent Technologies) and scanned immediately, using an Agilent High Resolution Microarray Scanner, at 5 micron resolution, 100% PMT. Scanned images were quantified using Feature Extraction software v 10.7.3.1 (Agilent Technologies) and statistical analysis of raw intensity data was performed in GeneSpring v12.1 (Agilent Technologies). Data for 3 independent biological replicate experiments were analysed. Data for each sample were normalized using a 75th percentile shift plus baseline normalized to the median of the related control sample for each biological replicate. Statistically significant (p < 0.05) differences of more than 10-fold between the two strains were identified in an unpaired t-test with Benjamini and Hochberg false discovery rate correction.

#### Real time RT-PCR

Bacteria were cultured as described for EM in CDM, 5.5 mM glucose, pH 7 to mid-log phase. Double volume of RNAprotect® bacteria reagent (Qiagen) was added. RNA was isolated and real-time RT- PCR performed as described previously to analyze expression of the first gene of the capsule operon, *cpsA* and competence gene *comX* [61]. The primer sequences for *comX* were: forward primer, 5′-TGT ATG AAG AAG TCC AAG GGA CTG T-3′, reverse primer, 5′-GTA AGC AGA GCA TGC CTT CTT G-3′ and probe 6-FAM-CCC ATA AAT GAA GGT AAT ATT-MGB_NFQ.

### Statistical analysis

GraphPad Prism v6.03 software for Windows (www.graphpad.com) was used to perform the statistical analysis with the unpaired t test with Welch’s correction. p ≤ 0.05, two-tailed, was considered significant.

### Nucleotide sequence accession numbers

The genome raw sequences of the Illumina runs are deposited in the short reads archive (http://www.ncbi.nlm.nih.gov/sra): 307.14, encapsulated (SRX485275) and 307.14, nonencapsulated (SRX485278). BioProject accession PRJNA241072 (http://www.ncbi.nlm.nih.gov/bioproject/241072).

## Results

### Clinical pneumococcal isolate 307.14 consists of two variants: one encapsulated and one nonencapsulated

**Figure 1:**
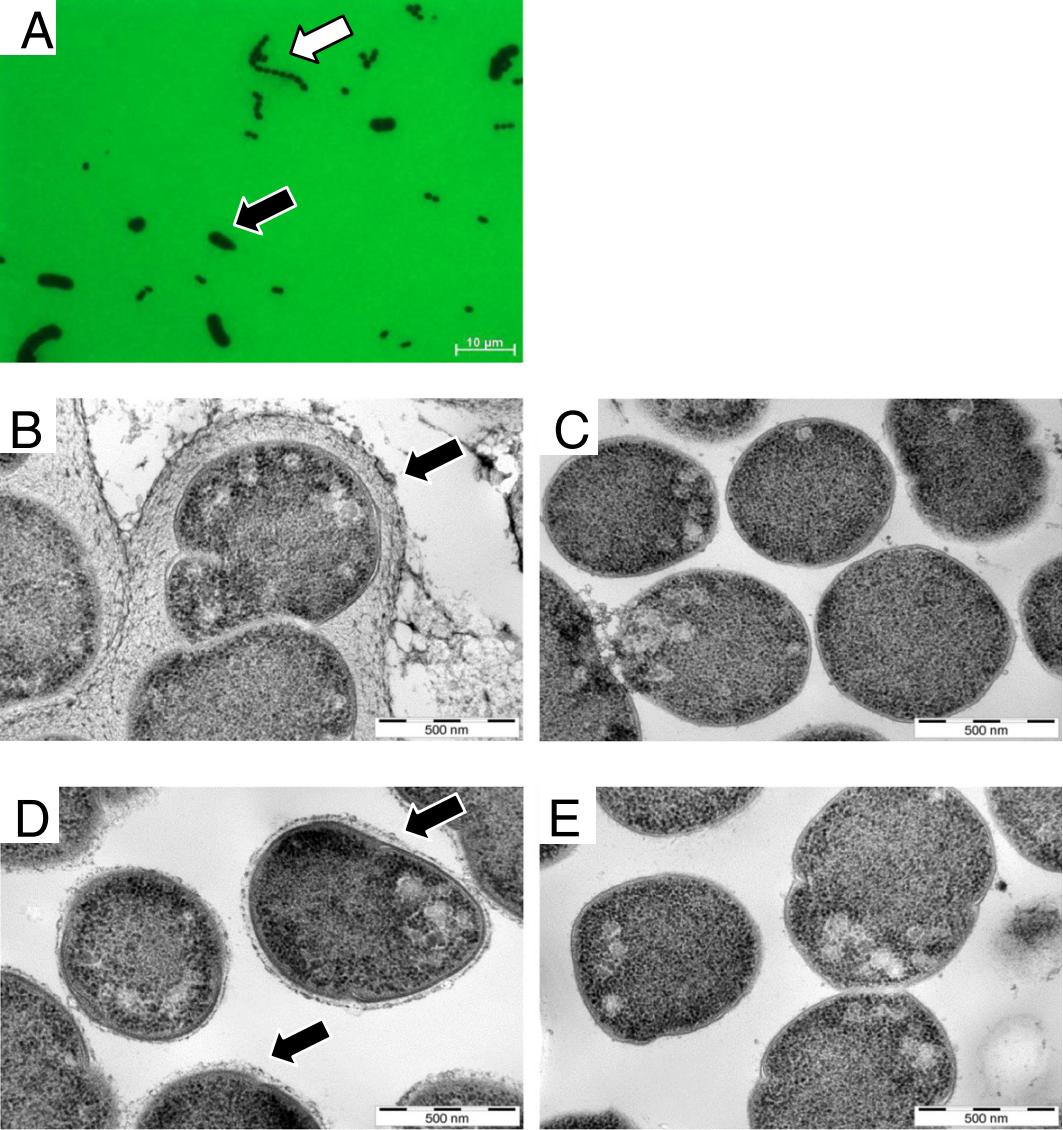
**Nasopharyngeal pneumococcal isolate 307.14 is composed of encapsulated and nonencapsulated variants and the phenotype is transferred with the capsule operon.** Strain 307.14 was cultured in CDM with 5.5 mM glucose and viewed by **(A)** fluorescence microscopy in the presence of FITC-dextran (100× objective). The black arrow indicates pneumococci expressing a large amount of capsule, the white arrow indicates pneumococci expressing no capsule. The two phenotypes were subcultured separately and viewed by transmission electron microscopy (EM). **(B)** 307.14 encapsulated pneumococci expressing a thick polysaccharide capsule (indicated by the black arrow), **(C)** 307.14 nonencapsulated pneumococci expressing no visible capsule. Capsule switch mutants were created in which the capsule operons of the two phenotypes were exchanged. **(D)** “307.14 nonencapsulated” in which the operon has been replaced with that of “307.14 encapsulated” (creating mutant 307.14 cap+), expressing a thin capsule (marked with a black arrow). **(E)** “307.14 encapsulated” in which the operon has been replaced with that of “307.14 nonencapsulated” (creating mutant 307.14 cap-), expressing no detectable capsule. EM pictures **(B)** to **(E)** were taken at a magnification of 53 000×.

On plating the nasopharyngeal isolate 307.14 onto CSBA plates, a mixture of large and small colonies was observed. The large colonies made up approximately 50% of the total and were serotyped as 18C (hereafter referred to as 307.14 encapsulated) whereas repeated attempts to type the small colonies led to the conclusion that they were nonencapsulated (hereafter referred to as 307.14 nonencapsulated). RFLP was performed on both phenotypes and confirmed that they were of the same genetic background (RFLP type 14, data not shown). Following subculture in CDM with 5.5 mM glucose, the bacteria were exposed to FITC-dextran. Figure 1A shows that by fluorescence microscopy two distinct sizes of bacteria could be observed. Transmission electron microscopy (EM) showed that while the encapsulated bacteria expressed a thick polysaccharide capsule (Figure 1B) the nonencapsulated expressed no visible capsule (Figure 1C).

To test the stability of the two phenotypes, each purified wild type variant was streaked onto CSBA plates and passaged six times over ten days on CSBA plates followed by culture in Lacks medium with 20 mM glucose on day 4 and day 9 of the experiment. Both phenotypes were stable as 307.14 encapsulated retained its capsule as determined by Quellung reaction and FITC-dextran exclusion assay and 307.14 nonencapsulated also maintained its phenotype over the repeated passaging (data not shown).

### Loss of capsule expression is due to a single point mutation in gene *cpsE*

Polymorphisms for each assembly of the Illumina reads with a quality score of at least 200× and that were not detected as assembly error in the remapping were further analyzed. 7 single base pair differences (SNPs) between 307.14 encapsulated and 307.14 nonencapsulated were revealed (Table 2). One was a switch from cytosine to guanine at position 1135 of the capsule gene *cpsE* predicted to cause a switch from arginine to glycine at position 379 of the protein.

**Table 2:** **Single nucleotide polymorphisms (SNPs) upon change of phenotype from 307.14 encapsulated to 307.14 nonencapsulated**

**Gene product (TIGR4, GenBank AE005672.3)**	**SNP position in gene (307.14)**	**Base change amino acid change**
Undecaprenylphosphate glucosephosphotransferase (CpsE) (SP_0350)	1135	C → G
		Arg → Gly
61 bp upstream ABC transporter, ATP-binding protein (SP_1282)	not applicable	A → G
		not applicable
NOL1/NOP2/sun family protein (SP_1402)	553	G → A
		Asp → Asn
Transposase, IS3 family, degenerate (SP_2018)	not applicable	T → G
		not applicable
dlt B protein (SP_2175)	449	A → G
		Stop → Trp
D-alanine-activating enzyme (SP_2176)	514	A → G
		Asn → Asp
Translation elongation factor Ts (SP_2214)	919	G → A
		Glu → Lys

The role of this SNP in *cpsE* in loss of capsule expression was confirmed by exchanging the capsule operons between the encapsulated and nonencapsulated phenotypes. When the capsule operon of 307.14 nonencapsulated was replaced by that of 307.14 encapsulated the expression of an 18C capsule was acquired as determined by serotyping and electron microscopy (Figure 1D). We named this mutant 307.14 cap + (Table 1). However, expression was lower than in the natural encapsulated strain: The mean thickness of the polysaccharide capsule of 307.14 encapsulated was 137 nm and for 307.14 cap + was 25 nm. Likewise, replacing the capsule operon of 307.14 encapsulated with that of 307.14 nonencapsulated caused it to lose capsule as shown by electron microscopy (Figure 1E) and it became nontypeable by Quellung reaction. We named this mutant 307.14 cap- (Table 1). The six other SNPs identified by whole genome sequencing were not transferred (confirmed by sequencing, see Additional file 1: Table S1) confirming that the SNP in *cpsE* is sufficient alone to change the capsule phenotype.

### Effect of loss of capsule expression on growth

**Figure 2:**
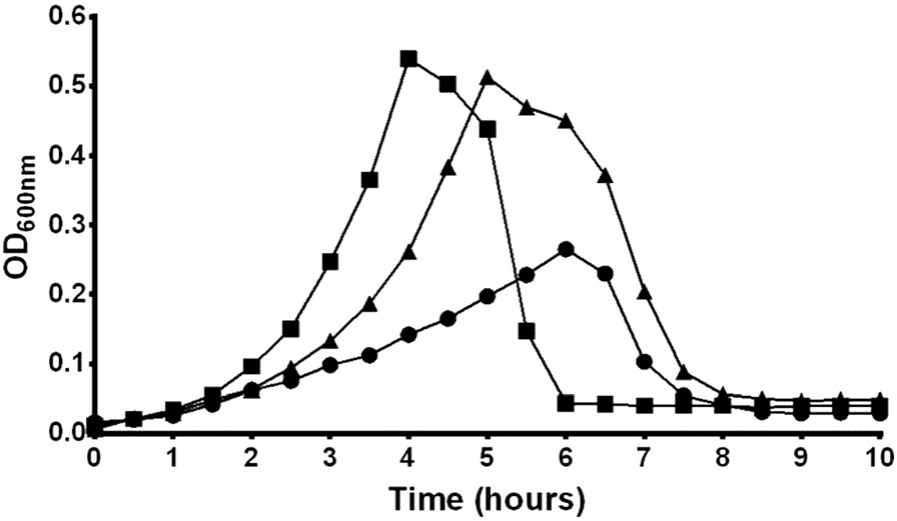
**Nonencapsulated variant of strain 307.14 has an advantage over the encapsulated variant in growth.** Growth was measured *in vitro* in CDM with 5.5 mM glucose by determining OD_600nm_ over 10 hours. Results show a representative of three independent experiments (see Additional file 1: Figure S1 for replicates). Wild type 307.14 encapsulated (●), wild type 307.14 nonencapsulated (■), laboratory mutant 307.14Δ*cps`:*Janus, nonencapsulated (▲).

Comparison of growth *in vitro* in a chemically defined medium (CDM) showed that the wild type 307.14 nonencapsulated, as well as the nonencapsulated laboratory mutant 307.14Δ*cps::*Janus, had a clear growth advantage over 307.14 encapsulated (Figure 2). The lag phase of growth was shorter and the maximal OD_600nm_ was higher for both of the nonencapsulated variants than the encapsulated (replicates shown in Additional file 1: Figure S1).

### Effect of loss of capsule on adherence and invasion

**Figure 3:**
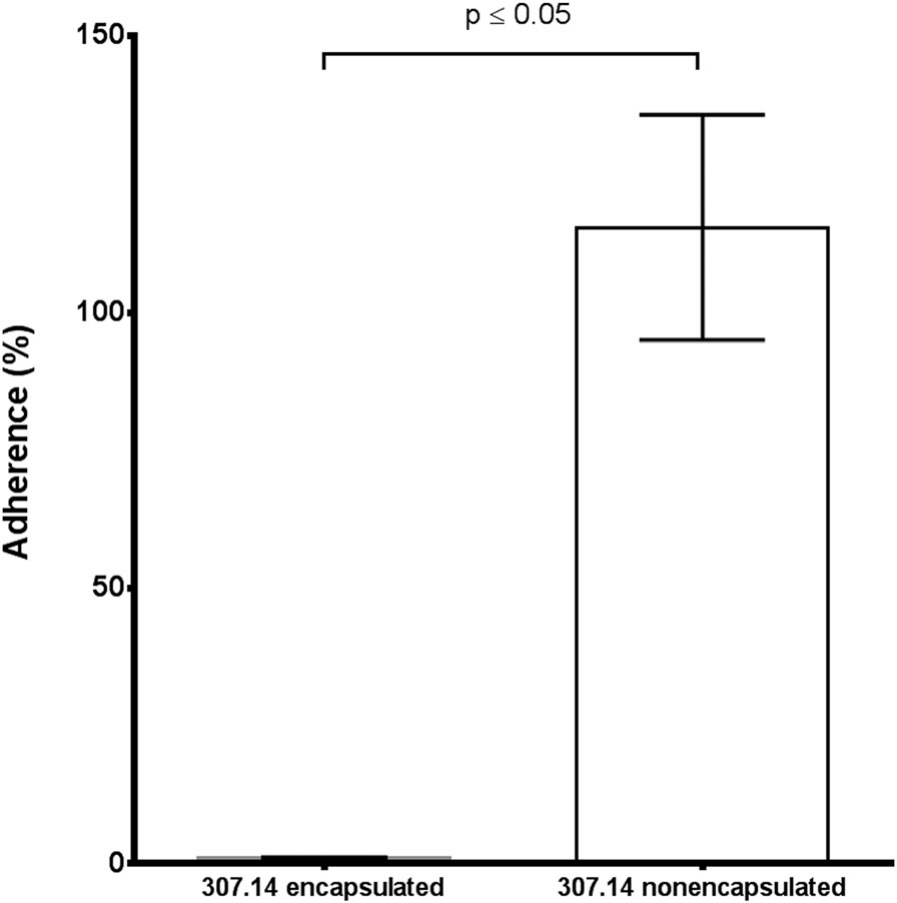
**Adherence of the two wild type variants to Detroit 562 human epithelial cells.** Means from three independent experiments, each performed in triplicate, are shown. Errors bars represent SEM. The statistical analysis was performed using unpaired t test with Welch’s correction.

For 307.14 encapsulated 1% of the inoculum adhered compared to 115% for 307.14 nonencapsulated. The relative value of adherent nonencapsulated 307.14 bacteria was presumably greater than 100% due to growth of the bacteria during the assay. This represents a 117-fold greater adherence for the nonencapsulated phenotype compared to the encapsulated (Figure 3). Invasion of the epithelial cells was also greater for the nonencapsulated phenotype: 0.22% for 307.14 nonencapsulated and 0.0012% for 307.14 encapsulated, a difference of 183-fold reflecting the difference in adherence.

### Antibiotic susceptibility

There were no significant differences in susceptibility of the two wild type variants to the antibiotics tested: ampicillin, benzylpenicillin, ceftriaxone, cephalothin, vancomycin, rifampicin, gentamicin, minocycline, tetracycline and colistin (Additional file 1: Table S3).

### Comparison of gene expression between encapsulated and nonencapsulated variants

**Figure 4:**
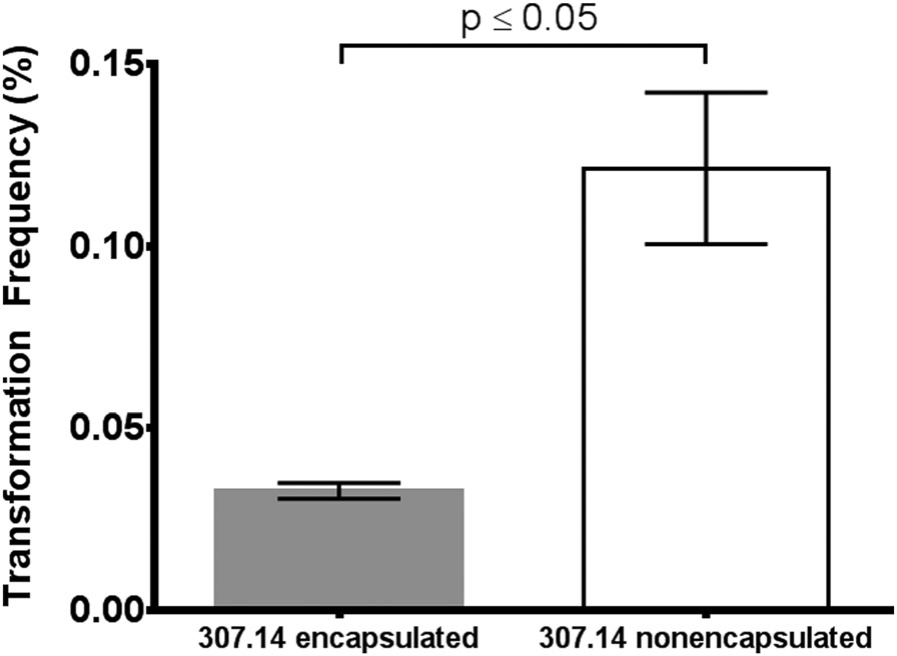
**Transformation frequencies of the two wild type variants.** Means from three independent experiments are shown. Error bars represent SEM. The statistical analysis was performed using unpaired t test with Welch’s correction.

Gene expression was investigated by microarray which showed that 307.14 encapsulated and 307.14 nonencapsulated expressed the genes of the capsule operon to an equal extent. This was confirmed for the first gene of the capsule operon, *cpsA*, by real-time RT-PCR (data not shown). However, seven other genes were upregulated in 307.14 nonencapsulated compared to 307.14 encapsulated between 11 and 34-fold (Table 3). For one of the genes, *comX*, expression was also determined by real-time RT-PCR by three independent experiments, each in triplicate. Comparing expression to that in the wild type encapsulated strain, a mean 3 fold higher expression was found in the wild type nonencapsulated strain, 35 fold higher in the 307.14 cap- mutant (differing from the wild type by only the SNP in *cpsE*) and 52 fold in the Janus mutant which lacks the entire capsule operon. Using the student t test with Welch’s correction these differences are not statistically significant, but the finding that nonencapsulated variants have a higher expression of *comX* than the encapsulated was consistent and in agreement with the microarray results. Strikingly, all seven genes identified by microarray were linked to competence, prompting us to compare the transformation frequencies between the variants. 307.14 encapsulated showed a mean transformation frequency of 0.0328% and 307.14 nonencapsulated of 0.1216% (Figure 4). This represents a 3.7-fold greater transformation frequency by the nonencapsulated variant compared to the encapsulated variant (p ≤ 0.05). Expression of no other genes differed significantly between the encapsulated and nonencapsulated phenotypes.

**Table 3:** **Microarray analysis showing upregulation of gene expression in 307.14 nonencapsulated versus 307.14 encapsulated phenotype**

**Gene**	**Function**	**Fold upregulation in nonencapsulated**
*comA*	competence	24
*comB*	competence	27
*comD*	competence	11
*comE*	competence	12
*comW*	competence	22
*comX*	competence	15
*orf51*	competence-induced bacteriocin B	34

## Discussion

Large and small pneumococcal colonies obtained from the nasopharynx of a child suffering from otitis media were due to two different patterns of capsule expression by one strain. The large colonies were due to bacteria expressing a thick capsule of serotype 18C and the small colonies were due to bacteria producing no detectable capsule. The difference was due to a single point mutation in the capsule gene *cpsE*. The resulting loss of capsule expression had clear consequences resulting in increased bacterial growth, adherence to epithelial cells and competence for genetic transformation. We speculate that the mutation occurred *in vivo* because the isolate contained an approximately 1:1 ratio of the encapsulated and nonencapsulated phenotypes. This is unlikely to have been achieved during the brief single laboratory culture before freezing the sample. We therefore conclude that the original colony derived directly from the patient contained a mixture of the encapsulated and nonencapsulated phenotypes.

Mutations in *cpsE* have been shown previously to lead to loss of capsule expression in clinical isolates. In 2012, Melchiorre *et al*., reported two pneumococcal isolates from patients with bacteraemic pneumonia. These isolates were nonencapsulated but with capsule operons very similar to those of serotype 7F strains. The isolates had two distinct point mutations in c*psE* both resulting in premature termination of transcription, CpsE which was truncated at the C terminus and loss of encapsulation in these two strains [62]. CpsE is the initial glycosyltransferase responsible for the addition of activated glucose-phosphate to the lipid carrier in the bacterial membrane [36-40]. Laboratory-generated *cpsE* knock-out mutants are also nonencapsulated [12]. Here it appears that an encapsulated and nonencapsulated phenotype can co-exist in the nasopharynx. It is also the first time a naturally-occurring mutation in *cpsE* leading to loss of capsule expression has been described in a serotype 18C strain. Unlike the SNP described by Melchiorre *et al*., the SNP described here does not result in a premature stop codon but rather an amino acid change from arginine to glycine. In addition, the location of the SNP differs from those described previously [34,35,62]. Our data suggest that the amino acid at position 379 in the cytoplasmic C terminal region of CpsE is critical for the function of the protein and therefore polysaccharide capsule production. *cpsE* is the first serotype specific gene following the conserved genes *cpsA* to *cpsD* [14,15]. However, there is high sequence similarity of *cpsE* gene throughout the serotypes [12,37-41,63,64] which raises the possibility that SNPs in *cpsE* may be a more general phenomenon to control capsule expression in other serotypes. This mechanism seems to be irreversible in contrast to the previously described mechanism of loss of capsule expression by spontaneous sequence duplication in the capsule operon [29,30]. Its irreversible nature, the apparent complete loss of capsule in the nontypeable phenotype and the fact that the two phenotypes did not vary in their expression of virulence gene such as *nanA*, *hylA*, *pspA* and *cbpA* differentiate the phenomenon we describe here from transparent/opaque phase variation [65,66].

We have shown before that loss of capsule affects phenotype, especially growth [23] and others have shown that a loss of capsule is associated with a gain in adherence to epithelial cells [67]. However, in our previous publication we used laboratory-generated capsule mutants in which the capsule operon was deleted and replaced by a Janus cassette. Here we show that in a nonencapsulated mutant that has lost its capsule naturally *in vivo* we also see the same effect i.e. an enhancement of growth.

Transformation is an important feature of the pneumococcus and does occur in its natural human environment [68]. Nonencapsulated strains are known to be more transformable than encapsulated strains [69] but our results indicate that this is not only due to a loss of the barrier of the capsule but also to an upregulation of genes involved in the competence pathway. Four temporally distinct expression profiles have been described in competence: early, late and delayed gene induction, and gene repression [70]. We noted with interest that the nonencapsulated phenotype had a higher expression of only the early competence genes compared to the encapsulated phenotype. Upregulation of early competence genes has been observed in tissue infections such as pneumonia and meningitis, but not sepsis, and may be linked to the pneumococci being in a biofilm-like state [71]. Whether the nonencapsulated phenotype described here is more often associated with biofilm than the encapsulated phenotype remains to be investigated.

We did not find a difference in antibiotic susceptibility between the two phenotypes. Fernebro *et al*. have shown that capsule expression reduces antibiotic-induced lysis however here we measured antibiotic resistance by Etest® and did not attempt to compare lytic responses [22].

A limitation of our study is that our isolate was from one patient at one timepoint. Although the fact that the two phenotypes were found at a ratio of approximately 1:1 suggests that they can co-exist *in vivo*, we do not know whether over time one phenotype would out-compete the other. We speculate that the nonencapsulated variant would have an advantage in colonization due to better growth and adherence and also be more able to take up foreign DNA (such as antibiotic resistance genes) giving it an advantage but we would need to make a study over time to determine this.

## Conclusions

We conclude that *cpsE* is critical for capsule expression in multiple serotypes. Mixtures of large and small colonies often seen in diagnostic laboratories and interpreted to be a mixture of strains could alternatively be a mixture of an encapsulated strain and its naturally-occurring nonencapsulated mutant. The link between loss of capsule expression and increased transformability may be due not only to a loss of the capsule barrier but also due to an upregulation of expression of genes of the competence pathway.

### Availability of supporting data

The data sets supporting the results of this article are included within the article and its additional files.

## Additional file

Additional file 1: Figure S1. Nonencapsulated variant of strain 307.14 has an advantage over the encapsulated variant in growth. This figure shows two replicates (A and B) of Figure 2. Growth was measured *in vitro* in CDM with 5.5 mM glucose by determining OD_600nm_ over 10 hours. Wild type 307.14 encapsulated (●), wild type 307.14 nonencapsulated (■), laboratory mutant 307.14Δ*cps:*Janus, nonencapsulated (▲). **Table S1:** Amplification and Sequencing Primers. **Table S2:** Preparation of the chemically defined medium (CDM). **Table S3:** Antibiotic susceptibilities. Minimal inhibitory concentrations (MIC) of the two *S. pneumoniae* 307.14 wild type variants to selected antibiotics determined by Etest® after 24 h and 48 h of incubation at 37°C and 5% CO_2_ atmosphere.

## Abbreviations

BHI: Brain heart infusion
bp: Base pairs
CDM: Chemically defined medium
CSBA: Columbia sheep blood agar
CSP-1: Competence-stimulating peptide
EM: Electron microscopy
FBS: Fetal bovine serum
FITC: Fluorescence isothiocyanate
MLST: Multi-locus sequence typing
PBS: Phosphate-buffered saline
RFLP: Restriction fragment length polymorphism
RHT: Ruthenium hexamine trichloride
SNP: Single nucleotide polymorphism
TSB: Tryptic soy broth
