# Supplementary material for: A point mutation in cpsE renders Streptococcus pneumoniae nonencapsulated and enhances its growth, adherence and competence
Source: BMC Microbiol. 2014 Aug 28;14:210. doi: 10.1186/s12866-014-0210-x (PMC4243769; doi:10.1186/s12866-014-0210-x)
Supplement: Additional file 1: Figure S1. — Nonencapsulated variant of strain 307.14 has an advantage over the encapsulated variant in growth. This figure shows two replicates (A and B) of Figure 2. Growth was measured in vitro in CDM with 5.5 mM glucose by determining OD600nm over 10 hours. Wild type 307.14 encapsulated (●), wild type 307.14 nonencapsulated (■), laboratory mutant 307.14Δcps:Janus, nonencapsulated (▲). Table S1: Amplification and Sequencing Primers. Table S2: Preparation of the chemically defined medium (CDM). Table S3: Antibiotic susceptibilities. Minimal inhibitory concentrations (MIC) of the two S. pneumoniae 307.14 wild type variants to selected antibiotics determined by Etest® after 24 h and 48 h of incubation at 37°C and 5% CO2 atmosphere. [file 12866_2014_210_MOESM1_ESM.pdf]

Supplementary Figure 1

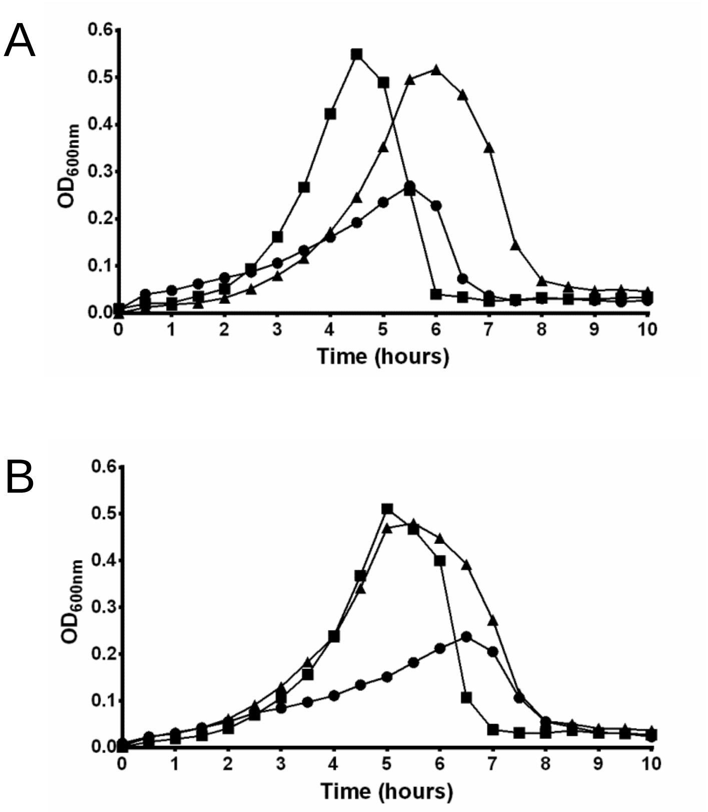

Figure S1 - Nonencapsulated variant of strain 307.14 has an advantage over the encapsulated variant in growth. This figure shows two replicates (A and B) of Figure 2. Growth was measured *in vitro* in CDM with 5.5 mM glucose by determining OD_{600nm} over 10 hours. Wild type 307.14 encapsulated (●), wild type 307.14 nonencapsulated (■), laboratory mutant 307.14Δ*cps::*Janus, nonencapsulated (▲).

Supplementary Table S1: Amplification and Sequencing Primers

Primer	Sequence (5' → 3')	Purpose	Reference
NCRspanFor3	AAA GGC TGC ACG GAC ATT G	RFLP	(1)
NCRspanRev3	CCG ATT TGC CAC TAG TGC GTA AGC	RFLP	(1)
rpsL_f	AGT GTA CAG GGA CGT GCT GAC AAA TGT TGC	PCR *rpsL*$_{K56T}$	Engel *et al*., 2014, submitted
rpsL_r	ATT GTA TAG CGG ATC TGG CAA TAC GTC ACG	PCR *rpsL*$_{K56T}$	Engel *et al*., 2014, submitted
dexBstart2	TTT CTC CCG TTT ATG ACA GCC CTA TGG	PCR Janus cassette	(2)
aliAend2	AAG ATT GGA CGC CCT GTA CGA GAT GT	PCR Janus cassette	(2)
aliAend1b	CTG GTT CAC TTG TAC CTT TAT TTC CT	Verification capsule operon orientation (3')	Morand, B. unpublished
cpsO_F1	CAA ATG GCC AAT TAG GAA CGG	Verification capsule operon orientation (3')	Morand, B. unpublished
RB_dexB_cspA_FA	CCT ATG CAA TGG GAC GAG AGC	Verification capsule operon orientation (5')	This study
RB_dexB_cpsA_RAB	TGC AAA GAG CGA CAC AGA GC	Verification capsule operon orientation (5')	This study
wchA_SNP_PCR_FOR	ATG GAA ATT GGT AGC AGT AAG TGT TTT G	Amplification SNP region *cpsE*	This study
wchA_SNP_PCR_REV	CCA TCT CTC ATA AAT ACA ACT TTA ACA GT	Amplification SNP region *cpsE*	This study
wchA_SNP_SEQ_FOR	CGT AAA GAT GGC GGA CCA GCT	Sequencing SNP region *cpsE*	This study
wchA_SNP_SEQ_REV	CCA CAT CTA ATT TTA CAA CAG CGT CAA	Sequencing SNP region *cpsE*	This study
SNP_2904_3_FOR	GCT GGT TCT CTC ATT CAA CTC TCA CTT	Amplification/ sequencing SNP	This study
SNP_2904_3_REV	TTT GAT ACT TCG AGT TCA GAC GTG ACA	Amplification/ sequencing SNP	This study
SNP_42678_FOR_1	AAA CCA GCT CTT CCA CAC TTG AAG T	Amplification/ sequencing SNP	This study
SNP_42678_REV_1	AGT CTC CTA AAT GCA CTT AGC CCT T	Amplification/ sequencing SNP	This study
SNP_80143_FOR_1	TAG AAG TAG CAG AGC CAA GCT TGA TTA	Amplification/ sequencing SNP	This study
SNP_80143_REV_1	GTT TTT GAG CTG TTT TGA CCG TCA ATT	Amplification/ sequencing SNP	This study
SNP_81628_FOR_1	TTT GCC TGG TAT GAA GTG TTG GTA AGT	Amplification/ sequencing SNP	This study
SNP_81628_REV_1	CAA GGC TAA ATT CTT CAG AGG AGG GAG	Amplification/ sequencing SNP	This study
SNP_86569_FOR_1	TAG CCT AAA ACT AAA CGA TCA AGC ACG	Amplification/ sequencing SNP	This study
SNP_86569_REV_1	TTC CTT CTT CGC CAA TAA TAG CTA CCT	Amplification/ sequencing SNP	This study
SNP_60490_FOR_1	ACC CTT AAA CTG TAG ATG GGC AAC AAA	Amplification/ sequencing SNP	This study
SNP_60490_REV_1	AAT CAA CTC AAC TGG CAG CCT ATC TAG	Amplification/ sequencing SNP	This study

1. **Hathaway LJ, Brugger S, Martynova A, Aebi S, Muhlemann K.** 2007. Use of the Agilent 2100 bioanalyzer for rapid and reproducible molecular typing of *Streptococcus pneumoniae*. J. Clin. Microbiol. **45:**803-809.

2. **Hathaway LJ, Brugger SD, Morand B, Bangert M, Rotzetter JU, Hauser C, Graber WA, Gore S, Kadioglu A, Muhlemann K.** 2012. Capsule type of *Streptococcus pneumoniae* determines growth phenotype. PLoS Pathog. **8:**e1002574.

Supplementary Table S2: Preparation of the chemically defined medium (CDM), according to (1), modified

Order of Addition to SB	Substance	Provider	Product Number	Stock Concentration	Amount stock for 1 l	Final Concentration	Remarks	Aliquots / Storage
1	Iron(II) sulfate heptahydrate	Merck KGaA	103965	10 mg/ml RWT1	0.5 ml	0.018 mM	freshly prepared	
2	Iron(III) nitrate nonahydrate	Merck KGaA	103883	10 mg/ml RWT1	0.1 ml	0.0025 mM	freshly prepared	
3	Potassium phosphate dibasic	Fluka	17835		0.2 g	1.1 mM	freshly prepared and dissolved in SB	
4	Potassium dihydrogen phosphate	Merck KGaA	104873		1 g	7.3 mM	freshly prepared and dissolved in SB	
5	Magnesium sulfate	Sigma-Aldrich	M7506	11.966 g/70 ml RWT1	2 ml	2.8 mM	dissolve in ice bath	sterile filtered, -20 °C
6	Manganese(II) sulfate monohydrate	Merck KGaA	105941	200 mg/40 ml RWT1	1 ml	0.03 mM		sterile filtered, -20 °C
7	L-Alanine	Sigma-Aldrich	A7627	2 g/100 ml RWT1	5 ml	1.1 mM		autoclaved, 4 °C
8	L-Arginine	Sigma-Aldrich	A5006	2 g/100 ml RWT1	5 ml	0.57 mM		autoclaved, 4 °C
9	L-Aspartic acid	Sigma-Aldrich	A9256	2 g/100 ml 0.2M HCl	5 ml	0.75 mM		sterile filtered, 4 °C
10	L-Phenylalanine	Merck KGaA	107256	2 g/100 ml 0.01M HCl	5 ml	0.61 mM	warm to dissolve	autoclaved, RT
11	L-Proline	Sigma-Aldrich	P0380	2 g/100 ml RWT1	5 ml	0.87 mM		autoclaved, 4 °C
12	L-Serine	Sigma-Aldrich	S4500	2 g/100 ml RWT1	5 ml	0.95 mM		autoclaved, 4 °C
13	L-Glutamic acid monosodium salt monohydrate	Sigma-Aldrich	G2834	2.544 g/100 ml RWT1	5 ml	0.68 mM		sterile filtered, 4 °C
14	L-Glutamine	Merck KGaA	100289	4 g/100 ml 1 M HCl	5 ml	1.4 mM		sterile filtered, 4 °C
15	Glycine	Merck KGaA	104201	2 g/100 ml RWT1	5 ml	1.3 mM		autoclaved, 4 °C

Order of Addition to SB	Substance	Provider	Product Number	Stock Concentration	Amount stock for 1 l	Final Concentration	Remarks	Aliquots / Storage
16	L-Threonine	Merck KGaA	108411	4 g/100 ml RWT1	5 ml	1.7 mM		autoclaved, 4 °C
17	L-Tryptophan	Sigma-Aldrich	T8941	2 g/100 ml 0.1 M NaOH	5 ml	0.49 mM	protect from light	sterile filtered, 4 °C
18	L-Tyrosine	Merck KGaA	108371	2.884 g/100 ml 1 M NaOH	5 ml	0.8 mM	protect from light	sterile filtered, 4 °C
19	L-Valine	Merck KGaA	108495	2 g/100 ml RWT1	5 ml	0.85 mM		autoclaved, 4 °C
20	L-Histidine monohydrochloride monohydrate	Sigma-Aldrich	H8125	2.7 g/100 ml RWT1	5 ml	0.64 mM		autoclaved, 4 °C
21	L-Isoleucine	Merck KGaA	105362	2 g/100 ml RWT1	5 ml	0.76 mM	warm to dissolve	autoclaved, 4 °C
22	L-Leucine	Sigma-Aldrich	L8912	2 g/100 ml RWT1	5 ml	0.76 mM	warm to dissolve	autoclaved, RT
23	L-Lysine monohydrochloride	Sigma-Aldrich	L5626	2.498 g/100 ml RWT1	5 ml	0.68 mM		autoclaved, 4 °C
24	L-Methionine	Merck KGaA	105707	2 g/100 ml RWT1	5 ml	0.67 mM		autoclaved, 4 °C
25	Folic acid	Sigma-Aldrich	F8758	30 mg/6 ml 1 M NaOH	0.16 ml	0.0018 mM	protect from light	sterile filtered, -20 °C
26	Biotin	Sigma-Aldrich	B4501	10 mg/100 ml RWT1	2 ml	0.00082 mM	warm to dissolve	sterile filtered, -20 °C
27	Nicotinamide	Sigma-Aldrich	N3376	40 mg/ 8 ml RWT1	0.2 ml	0.0082 mM		sterile filtered, -20 °C
28	D-Pantothenic acid hemicalcium salt	Sigma-Aldrich	P2250	80 mg/8 ml RWT1	0.2 ml	0.0084 mM		sterile filtered, -20 °C
29	4-aminobenzoic acid	Sigma-Aldrich	A9878	8 mg/8 ml RWT1	0.2 ml	0.0015 mM		sterile filtered, -20 °C
30	Pyridoxal hydrochloride	Sigma-Aldrich	P9130	40 mg/8 ml RWT1	0.2 ml	0.0049 mM	protect from light	sterile filtered, -20 °C
31	Pyridoxine hydrochloride	Sigma-Aldrich	P9755	40 mg/8 ml RWT1	0.2 ml	0.0049 mM	protect from light	sterile filtered, -20 °C
32	Pyridoxamine dihydrochloride	Fluka	P9380	40 mg/8 ml RWT1	0.2 ml	0.0041 mM	protect from light	sterile filtered, -20 °C
33	Thiamine hydrochloride	Sigma-Aldrich	T4625	40 mg/8 ml RWT1	0.2 ml	0.003 mM	protect from light	sterile filtered, -20 °C
34	Vitamin B_{12}	Sigma-Aldrich	V2876	5 mg/10 ml RWT1	0.2 ml	0.000074 mM	protect from light	sterile filtered, -20 °C

Order of Addition to SB	Substance	Provider	Product Number	Stock Concentration	Amount stock for 1 l	Final Concentration	Remarks	Aliquots / Storage
35	(-)-Riboflavin	Sigma-Aldrich	R4500	50 mg /+ 0.6 ml glacial acetic acid + 400 ml RWT1, dissolve, complement to 500 ml with RWT1	20 ml	0.0053 mM	warm to dissolve, protect from light	sterile filtered, -20 °C
36	Guanine hydrochloride	Sigma-Aldrich	51030	0.4 g/100 ml 1 M HCl	5 ml	0.11 mM		sterile filtered, RT
37	Adenine hemisulfate salt	Sigma-Aldrich	A9126	0.4 g/100 ml 1 M HCl	5 ml	0.11 mM		sterile filtered, RT
38	Uracil	Sigma-Aldrich	U0750	0.4 g/200 ml RWT1	10 ml	0.18 mM	warm to dissolve	autoclaved, RT
39	Calcium chloride	Merck KGaA	102083	202.4 mg/40 ml RWT1	1 ml	0.046 mM		sterile filtered, -20 °C
40	Sodium acetate	Merck KGaA	106268		2.7126 g	33.07 mM	dissolve in 100 ml SB	
41	di-Sodium hydrogen phosphate	Merck KGaA	106586		7.35 g	52 mM	dissolve in 200 ml SB	
42	Sodium dihydrogen phosphate monohydrate	Merck KGaA	106346		3.195 g	23 mM	dissolve in 100 ml SB	
43	Choline chloride	Sigma-Aldrich	C7527		1 g	7.2 mM	dissolve in 100 ml SB	
44	Sodium hydrogen carbonate	Merck KGaA	106329		2.5 g	30 mM	dissolve in 200 ml SB	
	**1) Measure pH and adjust to 6.85, 2) complement to 1000 ml SB, 3) filter sterilization and storage at 4 °C (use medium within three weeks of storage).**							
	**Add the following before use:**							
**Order of Addition to SB**	**Substance**	**Provider**	**Product Number**	**Stock Concentration**	**Amount stock for 1 l**	**Final Concentration**	**Remarks**	**Aliquots / Storage**
45	L-Cysteine hydrochloride	Sigma-Aldrich	C7477	0.75 g/10 ml RWT1	10 ml	4.8 mM		sterile filtered, 4 °C
46	β-Nicotinamide adenine dinucleotide hydrate	Sigma-Aldrich	N1511	10 mg/ml RWT1	0.25 ml	0.0038 mM		sterile filtered, 4 °C
47	Copper(II) sulfate pentahydrate	Sigma-Aldrich	C7631	10 mg/10 ml RWT1	0.6 ml	0.0024 mM		sterile filtered -20 °C
48	Manganese(II) sulfate monohydrate	Merck KGaA	105941	125 mg/5 ml	1 ml	0.15 mM		sterile filtered -20 °C

Preparation of the Sørenson phosphate buffer (pH 7) for 1 liter CDM

**Solution A**		**Final Concentration**
Potassium dihydrogen phosphate (Merck 104873)	3.632 g in 400 ml RWT1	26 mM
**Solution B**		
di-Sodium hydrogen phosphate dihydrate (Merck 106580)	8.316 g in 700 ml RWT1	41 mM
**Sørensen phosphate buffer (pH 7)**		
Solution A: 392 ml<br>Solution B: 608 ml		

RWT1 = ultra-pure distilled water, SB = Sørensen phosphate buffer, RT = room temperature

1. **van de Rijn I, Kessler RE.** 1980. Growth characteristics of group A streptococci in a new chemically defined medium. Infect. Immun. **27:**444-448.

Supplementary Table S3: Antibiotic susceptibilities. Minimal inhibitory concentrations (MIC) of the two *S. pneumoniae* 307.14 wild type variants to selected antibiotics determined by Etest® after 24 h and 48 h of incubation at 37 °C and 5 % CO_2 atmosphere.

		MIC of 307.14 encapsulated		MIC of 307.14 nonencapsulated	
Antibiotic	Etest® code	24 h	48 h	24 h	48 h
Ampicillin	AM	0.016	0.016	0.016	0.016
Benzylpenicillin	PG	0.016	0.016	0.016	0.023
Ceftriaxone	TX	0.012	0.016	0.016	0.016
Cephalothin	CE	0.064	0.064	0.25	0.25
Vancomycin	VA	0.25	0.25	0.25	0.25
Rifampicin	RI	0.016	0.016	0.016	0.016
Gentamicin	GM	6 (8)[1]	8	2 (3)[1]	3
Minocycline	MC	0.094	0.125	0.094	0.125
Tetracycline	TC	0.094	0.094	0.125	0.094
Colistin[2]	CO	>256	>256	>256	>256

[1]Heterogeneous MIC value; minor number of colonies shows a slightly higher antibiotic resistance (this MIC value is given in brackets).

[2]The antibiotic colistin was used as a reference antibiotic. Gram-positive aerobic cocci are generally resistant to colistin (1).

1. **Falagas ME, Kasiakou SK.** 2005. Colistin: the revival of polymyxins for the management of multidrug-resistant gram-negative bacterial infections. Clin. Infect. Dis. **40:**1333-1341.
